# The high *FKBP1A* expression in WBCs as a potential screening biomarker for pancreatic cancer

**DOI:** 10.1038/s41598-024-58324-z

**Published:** 2024-04-03

**Authors:** Papitchaya Watcharanurak, Apiwat Mutirangura, Vitavat Aksornkitti, Narumol Bhummaphan, Charoenchai Puttipanyalears

**Affiliations:** 1 Faculty of Medical Technology, Prince of Songkla University, Hat Yai, Songkhla, 90110 Thailand; 2 Department of Anatomy, Faculty of Medicine, Chulalongkorn University, 1873 Rama IV Road, Pathumwan, Bangkok, 10330 Thailand; 3 Center of Excellence in Molecular Genetics of Cancer and Human Diseases, Department of Anatomy, Faculty of Medicine, Chulalongkorn University, Bangkok, 10330 Thailand; 4 College of Public Health Sciences, Chulalongkorn University, Sabbasastravicaya Building, Phayathai Road. Wangmai, Pathumwan, Bangkok, 10330 Thailand

**Keywords:** Pancreatic cancer, Biomarkers, White blood cells, Real time polymerase chain reaction, Biotechnology, Cancer, Molecular biology, Biomarkers, Medical research

## Abstract

Given the limitation of current routine approaches for pancreatic cancer screening and detection, the mortality rate of pancreatic cancer cases is still critical. The development of blood-based molecular biomarkers for pancreatic cancer screening and early detection which provide less-invasive, high-sensitivity, and cost-effective, is urgently needed. The goal of this study is to identify and validate the potential molecular biomarkers in white blood cells (WBCs) of pancreatic cancer patients. Gene expression profiles of pancreatic cancer patients from NCBI GEO database were analyzed by CU-DREAM. Then, mRNA expression levels of three candidate genes were determined by quantitative RT-PCR in WBCs of pancreatic cancer patients (N = 27) and healthy controls (N = 51). ROC analysis was performed to assess the performance of each candidate gene. A total of 29 upregulated genes were identified and three selected genes were performed gene expression analysis. Our results revealed high mRNA expression levels in WBCs of pancreatic cancer patients in all selected genes, including *FKBP1A* (*p* < 0.0001), *PLD1* (*p* < 0.0001), and *PSMA4* (*p* = 0.0002). Among candidate genes, *FKBP1A* mRNA expression level was remarkably increased in the pancreatic cancer samples and also in the early stage (*p* < 0.0001). Moreover, *FKBP1A* showed the greatest performance to discriminate patients with pancreatic cancer from healthy individuals than other genes with the 88.9% sensitivity, 84.3% specificity, and 90.1% accuracy. Our findings demonstrated that the alteration of *FKBP1A* gene in WBCs serves as a novel valuable biomarker for patients with pancreatic cancer. Detection of *FKBP1A* mRNA expression level in circulating WBCs, providing high-sensitive, less-invasive, and cost-effective, is simple and feasible for routine clinical setting that can be applied for pancreatic cancer screening and early detection.

## Introduction

According to GLOBOCAN 2020, pancreatic cancer is the seventh leading cause of cancer mortality worldwide. Mortality is estimated to continue rising over the next few decades, becoming the second most common cancer-related death by 2040^1^. Pancreatic cancer is an aggressive malignancy with very poor prognosis, represented by an mortality-to-incidence ratio (MIR) of about 94%^2^. Patients with pancreatic cancer are commonly diagnosed at an advanced stage, with only 10% detected at an early stage^3^. This is due to patients with pancreatic cancer having obscure symptoms with most cases asymptomatic at the early stage, leading to tumor progression and subsequent non-responsiveness to curative treatment^4,5^. Compared to other tumors, the five-year survival rate of pancreatic tumors is very low at less than 10%. However, if patients can be detected at early-stage and undergo surgical resection, they would have a 5-year survival rate more than ten-fold higher compared to patients with advanced stage or metastasis^6–10^. At the present time, there are no clinical approaches to screen or diagnose early pancreatic cancer, especially in asymptomatic patients^11^. As a result, the discovery of potential methods or biomarkers for screening and early detection is crucial to improve cancer prognosis, reduce mortality, and enhance the chance for effective treatment.

The diagnosis of pancreatic cancer is mainly operated by biopsy and imaging tests such as endoscopy, computed tomography (CT) scan, and magnetic resonance imaging (MRI). Although these examinations play important roles in clinical practice, they are invasive and possess unsatisfied sensitivity in the early stage^12–14^. The identification of a blood-based biomarker can offer critical information in pancreatic cancer detection. Currently, serum carbohydrate antigen 19‐9 (CA19‐9) is the only biomarker approved by United States Food and Drug Administration for pancreatic cancer diagnosis and monitoring^15^. However, CA19‐9 lacks tumor specificity. High CA19-9 levels can also be indicative of different types of cancer such as esophageal, stomach, colon, liver, bile duct, and ovary^16–20^. CA19-9 is not currently considered as a screening test for pancreatic cancer patients due to its low positive predictive value^21,22^.

Increasing studies are focusing on a cancer biomarker derived from circulating blood cells which can be an effective and less-invasive method^23–25^. Our recent studies have revealed a mechanism by which cancer cells leave cytokine-like secretions in the surrounding WBCs which result in the alteration of WBC’s epigenetic and gene expression profiles. This mechanism has appeared in several types of cancer, including breast cancer, hepatocellular carcinoma, head and neck squamous cell carcinoma, and colon cancer^26–29^. Based on this knowledge, this study aimed to evaluate promising novel biomarkers for pancreatic cancer screening and detection by identifying differences in gene expression profiles of WBCs. The results of this study would be beneficial for future screening or for diagnostic purposes in pancreatic cancer patients.

## Results

### Bioinformatics

**Figure 1:**
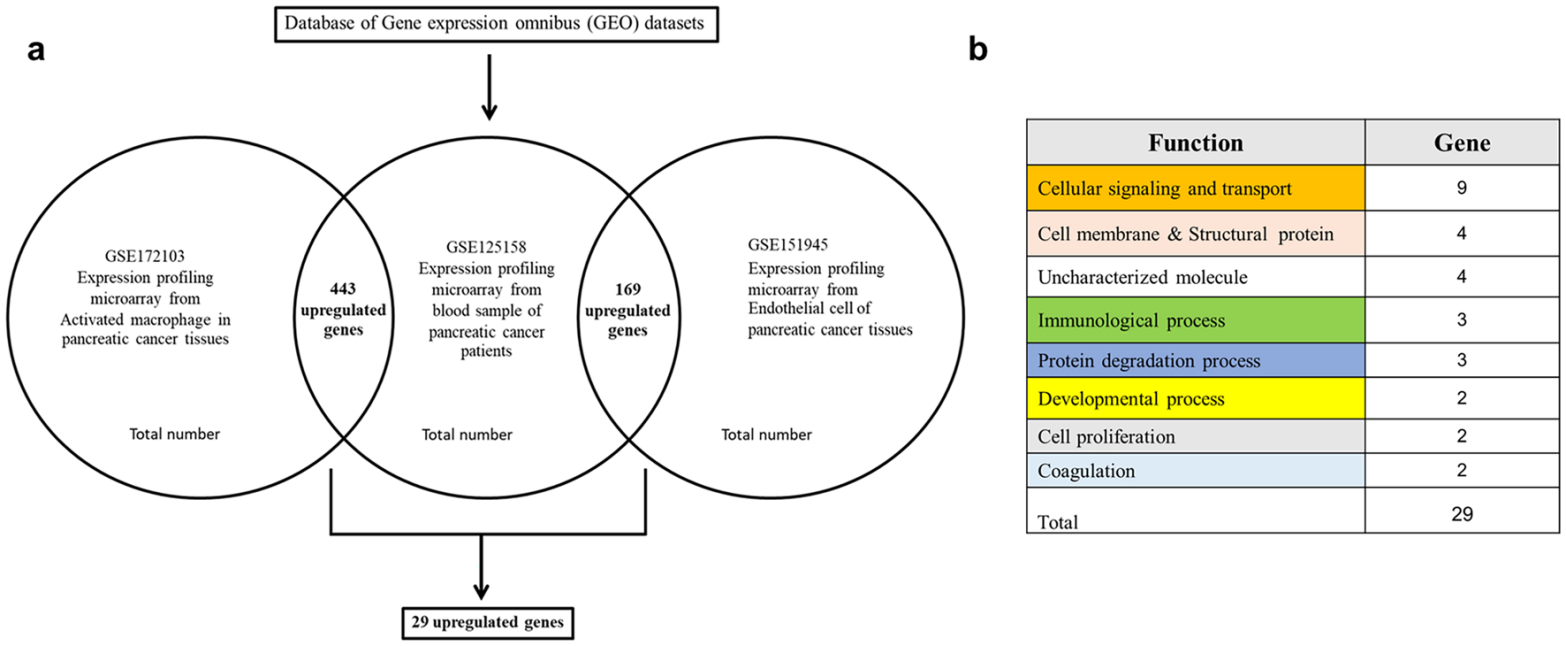
Summary of the experiment. Each gene expression from each dataset including GSE172103, GSE125158, and GSE151945 was analyzed by a “Connection Up and Down-Regulation Expression Analysis of Microarrays (CU-DREAM) to evaluate the intersection genes. (**a**) Venn diagram based on the overlapping number of upregulated genes among microarray data groups indicated 29 genes indicating upregulated differentially expressed genes (DEG); (**b**) Total 29 candidate genes determined by biological process from gene ontology as represented in the table according to function of mechanism.

The expression profiling microarray corresponding to pancreatic cancer was extracted to screen for upregulated genes. Data from the GEO dataset including GSE172103, GSE125158, and GSE151945 were analyzed by the CU-DREAM program. By intersecting data across the 3 datasets, a total number of 29 upregulated genes were obtained (Fig. 1A). The functions of identified genes were then classified according to their biological functions as shown in Fig. 1B. Three candidate genes with the highest significant *p*-values in cell signaling and transport, immunological process, as well as protein degradation process were selected, consisting of *FKBP1A* (*p* = 2.88 × 10^–6^), *PLD1* (*p* = 2.82 × 10^–5^), and *PSMA4* (*p* = 4.5 × 10^–4^). The details of the biological function of the genes are provided in supplement information 1.

### Upregulation of *FKBP1A*, *PLD1, *and *PSMA4* gene expression

**Figure 2:**
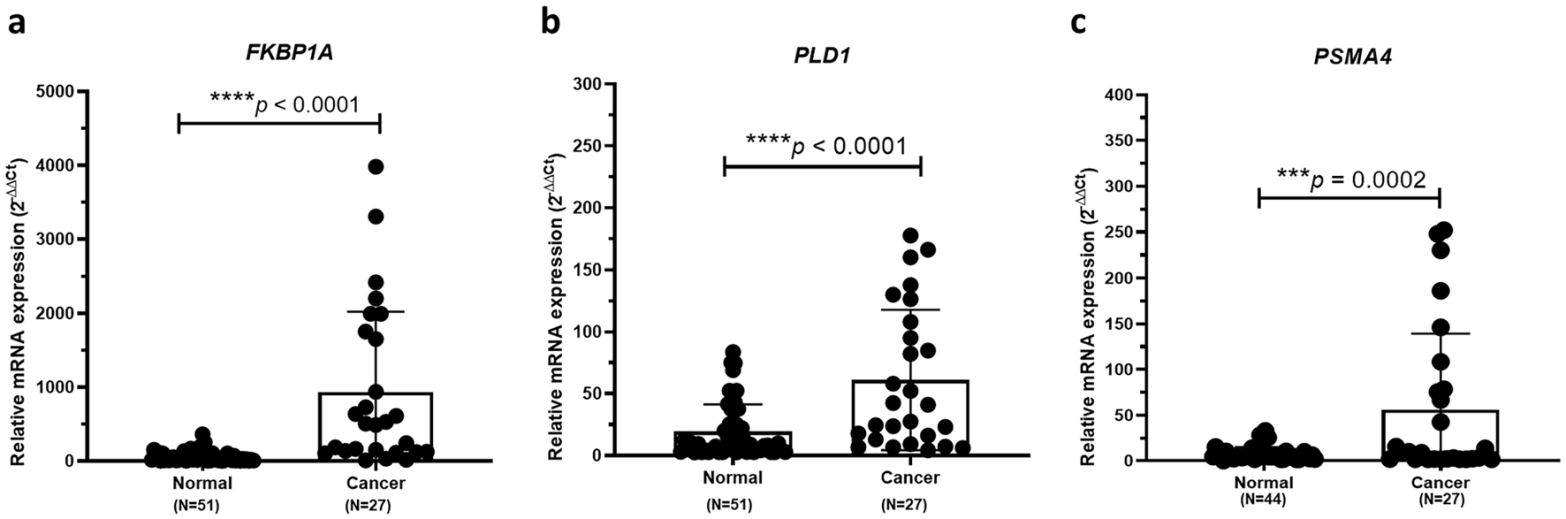
qRT-PCR results were performed in cDNA from all blood samples. The relative mRNA expression value of each gene was compared to the housekeeping gene (*GAPDH*) within the healthy control group and pancreatic cancer group and shown as mean ± SD. The Ct values in the pancreatic group were significantly increased compared to the healthy controls with *p*-value < 0.0001 in *FKBP1A* (**a**) and *PLD1* (**b**), and *p*-value = 0.0002 in *PSMA4* (**c**).

To investigate gene expression in WBC-associated pancreatic cancer, mRNA expression levels of three selected genes including *FKBP1A*, *PLD1*, and *PSMA4* were evaluated using qRT-PCR. We found that these genes were differentially expressed in cancer. The quantification showed that mRNA levels of all candidate genes were significantly higher in pancreatic cancer samples compared to controls (*p* < 0.0001 in *FKBP1A, p* < *0.0001* in *PLD1, and p* = *0.0002* in *PSMA4*). Interestingly, the mRNA expression levels of *FKBP1A* in some pancreatic cancer patients were a thousand-fold higher than in control samples (Fig. 2).

### Comparison of gene expression and stages of tumor

**Figure 3:**
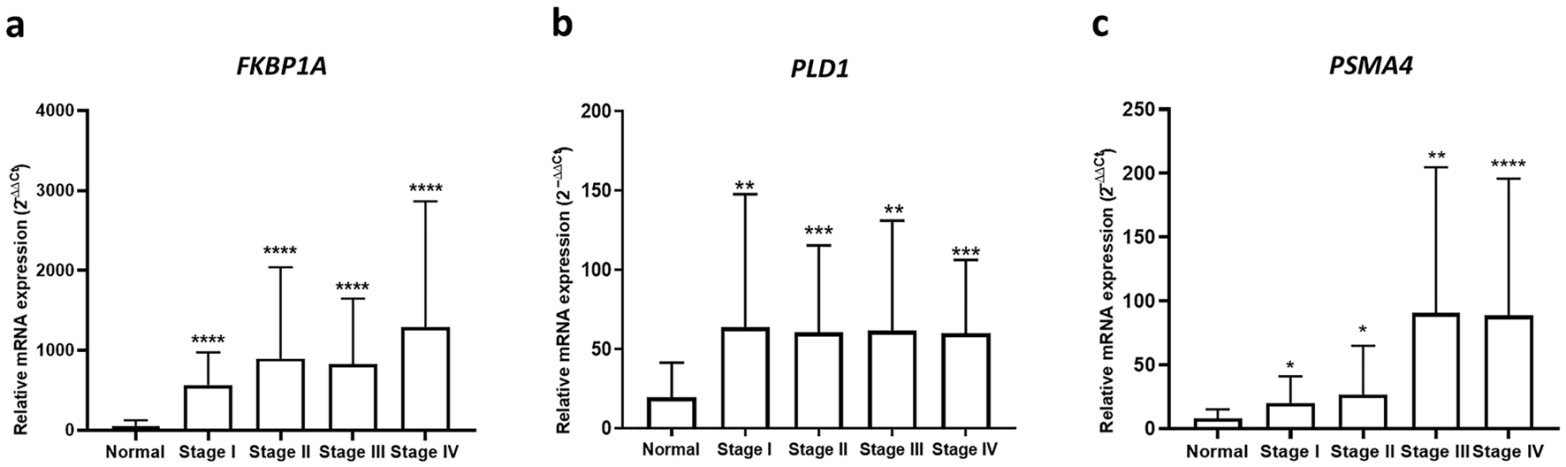
Additional analysis in stages of mRNA expression in *FKBP1A* (**a**), *PLD1* (**b**), and *PSMA4* (**c**) genes. The figures show the statistically significance (*p*-value) of all gene expressions when pairing between stage I, II, III, and IV compared with normal control (all using t-test).

Next, we assessed whether the expression of selected genes (*FKBP1A, PLD1,* and *PSMA4*) was correlated to the tumor stage. The mRNA expression level of the indicated gene was compared between control and pancreatic cancer samples in each stage (I-IV). As shown in Fig. 3, significant increases in mRNA levels of all indicated genes were detected at all stages of the tumor, however; there was no statistical significance between tumor stages. In the early stage, *FKBP1A* exhibited the highest significant increase when compared to controls (*p* < 0.0001). In addition, the mRNA expression level of *FKBP1A* and *PSMA4* revealed statistically significant increases in the late stage (*p* < 0.0001).

### ROC analysis

**Figure 4:**
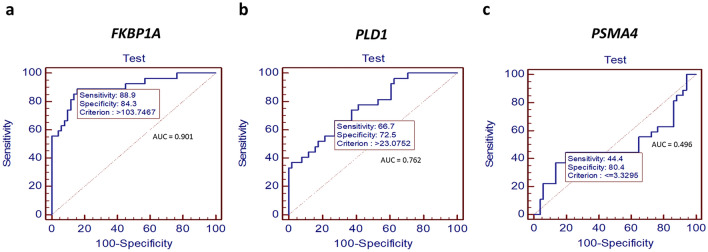
The ROC graph and the result of sensitivity and specificity in each gene including; *FKBP1A* (**a**), *PLD1* (**b**), and *PSMA4* (**c**). The sensitivity and specificity of *FKBP1A* gene showed the greatest value compared to other genes with 88.9% sensitivity, 84.3% specificity and AUC data = 0.901.

To evaluate the pancreatic cancer detection efficiency of the candidate genes in terms of sensitivity, specificity, and accuracy, receiver operating characteristic (ROC) curve analysis was performed as presented in Fig. 4. The results showed that the mRNA expression level of the *FKBP1A* gene in WBCs provided excellent accuracy (AUC = 0.901) with a sensitivity of 88.9% and a specificity of 84.3%. For *PLD1* and *PSMA4* genes, the sensitivity was 66.7% and 44.4%, and the specificity was 72.5% and 80.4%, respectively.

### *FKBP1A* validation

We further tested other 18 WBC samples (April 2021 to April 2022), including 7 cases of pancreatic cancers (1 case for stage 1, 1 case for stage 2, and 5 cases for stage 3), and 11 cases of healthy normal. All samples were examined by double-blind test to validate the predictive ability of *FKBP1A* expression. With relative expression of 103.7467, the accuracy rate of *FKBP1A* in detecting the presence of pancreatic cancer was 88.89%. The result was presented in supplement information 2.

### *FKBP1A* expression is tumor specific

**Figure 5:**
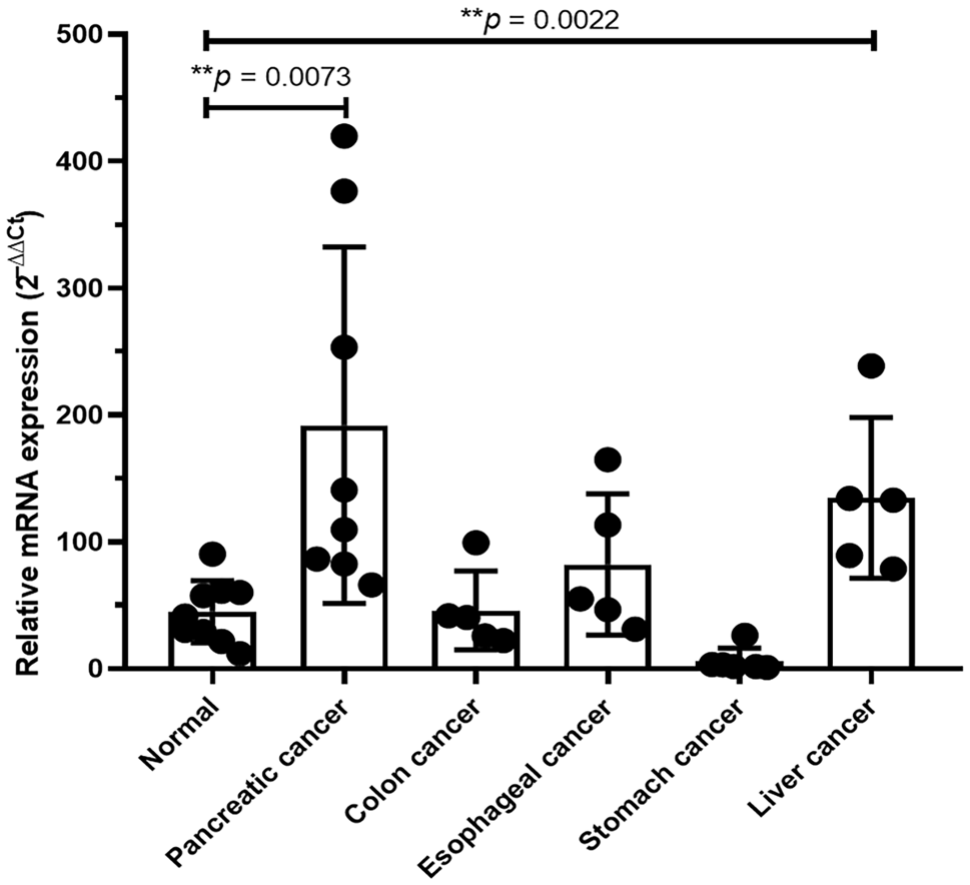
The specification of *FKBP1A* gene expression in pancreatic cancer. mRNA level of *FKBP1A* gene was significantly increased in pancreatic cancer compared with to normal controls and other cancer-related digestive system including of colon, esophageal, stomach, and liver cancer (*p* = 0.0073). The mRNA level, however, was highly expressed in liver cancer (*p* = 0.0022) compared to normal control.

We also tested the specificity of the *FKBP1A* gene by comparing mRNA levels with other cancer-related digestive systems, including pancreatic cancer (N = 8), esophageal cancer (N = 5), stomach cancer (N = 5), colon cancer (N = 5), and liver cancer (N = 5). In pancreatic cancer, the results revealed a significant increase of *FKBP1A* mRNA expression (*p* = 0.0073), while in other cancers, except liver cancer (*p* = 0.0022), the mRNA level was unchanged (Fig. 5).

## Discussion

Pancreatic cancer is typically found during the late stage and is difficult to diagnosis early due to unspecified symptoms and the inability to identify through MRI imaging. Currently, the screening marker CA19-9 delivers unsatisfactory efficiency prompting the need for a new molecular biomarker for pancreatic cancer. In this current work, we integrated bioinformatics analysis (CU-DREAM) with a molecular approach (RT-qPCR) to demonstrate the *FKBP1A* gene as a novel molecular biomarker for pancreatic cancer detection. At the mRNA level, *FKBP1A* gene expression in WBCs of pancreatic cancer patients was significantly higher than normal and showed satisfactory sensitivity (88.9%) and specificity (84.3%) with an AUC of 0.901. Multiple studies have postulated about the ability of cancer cells to produce cytokine and chemokine-like secretion molecules which affect gene expression changes in blood circulating immune cells, including WBCs, contributing to the progression of various forms of cancer and impacting outcomes^31–34^. Recent works have shown that the levels of mRNA and protein expression of PBMCs exhibited changes in both hepatocellular carcinoma and non-small cell lung cancer^27,35^. In line with this previous work, our findings also described the mechanism of cancer cytokine signaling in molecule-regulated gene expression of immune cells in pancreatic cancer. In addition, co-culture studies revealed that secretion molecules from cancer cells could alter epigenetics via DNA methylation in blood immune cells in several types of cancers such as head and neck, colorectal, and breast^26,28,29^.

In our comparison between the normal and early-stage cancer groups, *FKBP1A* showed the highest significant *p*-value (*p* < 0.0001) among all candidate genes (*PLD1*, *p* = 0.0078, *PSMA4*, *p* = 0.0165). Although a small amount of tumor cells was present in the early stage, thousands of blood-circulating immune cells which consist of WBCs and PBMCs can be activated through cancer secretion^28,29^. This interaction between cancer and immune cells increases the opportunity and sensitivity for cancer detection, especially at an early stage. Our results suggested that *FKBP1A*, which showed the best performance, could be applied for early detection of pancreatic cancer. Moreover, *FKBP1A* and *PSMA4* mRNA expression levels tended to be higher in advanced pancreatic cancer stages. This might be due to increased cancer cell growth and proliferation during advanced tumor stages which results in an increase in cancer immune cells interaction. Therefore, these increased levels of gene expression in the immune cells are promising in developing highly predictive cancer biomarkers.

*FKBP1A*, belonging to FKBPs family, is a cis–trans prolyl isomerase (PPIase) enzyme^36^. This protein functions as a receptor of immunosuppressive drugs which can bind to the immunosuppressants FK506 and rapamycin. It plays an essential role in immune cell signaling. It can also interact with many intracellular signal transduction proteins including type I TGF-beta receptor^37,38^. Evidence has shown that *FKBP1A* participates in multiple malignancies such as head and neck, breast, lung, and liver^39–41^. In this study, the higher *FKBP1A* gene expression level was identified in WBCs of pancreatic cancer patients compared to controls, indicating that *FKBP1A* is involved in the cell-to-cell communication between immune cells and pancreatic cancer. Moreover, our experiments revealed that the tumor specific property that elevated *FKBP1A* gene expression was not only found in pancreatic cancer WBCs but also in hepatic cancer. Consistent with our results, a recent study revealed that protein expression of *FKBP1A* was found in hepatocellular carcinoma tissues and its expression was correlated with stage, grade, and metastasis of the tumor. In addition, a positive correlation between *FKBP1A* expression and immune cells such as B cells, CD8+ T cells, and CD4+ T cells was observed^41^. In contrast, downregulated *FKBP1A* in breast cancer tissues is associated with poor prognosis and increased resistance to chemotherapy^40^. Upregulation of *FKBP1A* was found to diminish cancer cell growth in glioblastoma, a type of brain tumor^42^. The limitations of this research included the absence of in vitro study such as co-cultured techniques because the interaction between cancer and the immune cell process occurs from multiple factors that cannot reproduced by in vitro studies. Another limitation is the small sample population that did not include subjects with pancreatitis. Due to the nature characteristics of pancreatic cancer, the most of patients were diagnosed at very late stage. However, the result from our study demonstrated *FKBP1A *as a clinically validated biomarker in early and advanced stage.

In summary, our study is the first to document mRNA expression levels of *FKBP1A* in WBCs as a potential biomarker for pancreatic cancer detection and show that elevated *FKBP1A* mRNA expression can distinguish early-stage pancreatic cancer patients from healthy controls. These findings also shed light on the pathogenesis involving immunological regulation in pancreatic cancer. Utilizing the differential *FKBP1A* gene expression in circulating WBCs of pancreatic cancer patients is simple, less invasive, and exhibited high sensitivity in our study, when compared to the conventional serum biomarker CA19-9 and imaging methods (MRI). This approach is feasible in clinical practice and can be applied for mass screening and early detection for pancreatic cancer in the near future.

## Materials and methods

### Bioinformatics analysis

Three Gene Expression Omnibus (GEO) datasets were selected from the National Center for Biotechnology Information (NCBI) database (https://www.ncbi.nlm.nih.gov/geo/), including GSE172103, GSE125158 and GSE151945. The GSE125158 gene expression microarray, which included blood samples from pancreatic cancer patients, was compared with GSE172103 which is the expression profile of activated macrophages in pancreatic cancer tissues and GSE151945 which is the expression profile of endothelial cells in pancreatic cancer tissues. GEO datasets were analyzed for the gene expression levels using the CU-DREAM (Connection Up- or Down-Regulation Expression Analysis of Microarrays Extra, website: http://pioneer.netserv.chula .ac) program and calculated *p*-values and odds ratios. All upregulated genes from GSE125158, GSE172103, and GSE151945 were analyzed. We manually observed the gene function of all upregulated genes (Fig. 1). Three candidate genes with significant *p*-values were then selected from the list of upregulated genes to observe gene expression in WBCs of pancreatic cancer patients.

### Study population

This cross-sectional case–control study was performed at the Faculty of Medicine, Chulalongkorn University. All samples were recruited from King Chulalongkorn Memorial Hospital composed of the experimental set collected during March 2017 to March 2021 including 51 healthy normal and 27 pancreatic cancer patients, and the validation set collected during April 2021 to April 2022 including 11 healthy normal and 7 pancreatic cancer patients. All cancer cases were staged according to the revised Tumor, Node and Metastasis (TNM) classification criteria by a pathologist. Healthy normal samples were collected from the patients without a family history of cancer, immune disorders, jaundice and chronic diseases. The demographic data of all samples is presented in Table 1. All participants in this study were of Asian descent and provided their written informed consent to participate in this study.

**Table 1:** The demographic data of experimental set consisted of healthy normal controls (N = 51) and pancreatic cancer (N = 27) samples which divided by stage I to stage IV in males and females.

Sample	Age	Gender	
	Average ± SD	Male	Female
Healthy normal (N = 51)	62.41 ± 9.56	23 (45.10%)	28 (54.90%)
Pancreatic cancer (N = 27)	64.56 ± 8.01	14 (51.85%)	13 (48.15%)
- Stage I (N = 3)	57.33 ± 9.71	1 (33.33%)	2 (66.67%)
- Stage II (N = 11)	64.55 ± 8.43	8 (72.73%)	3 (27.27%)
- Stage III (N = 7)	64.43 ± 5.06	3 (42.86%)	4 (57.14%)
- Stage IV (N = 6)	68.33 ± 8.59	2 (33.33%)	4 (66.67%)

The average age of each group was shown as mean ± SD. The staging of pancreatic cancer showed 3 samples in stage I, 11 samples in stage II, 7 samples in stage III, and 6 samples in stage IV.

### Sample size calculation

We used the preliminary results from GSE172103, GSE125158 and GSE151945 to find the appropriate sample size with the following formula^26,27^:

$${\text{N}} = \left[ {{{\left( {{{\text{Z}}_{{{\upalpha /2}}}} + {{\text{Z}}_{\upbeta }}} \right)}^2}\left( {{{\upsigma }^2}_{\text{d}}} \right)} \right]/{\left( {{{\bar{\text{x}}}_{\text{d}}}} \right)^2}$$

N = sample size, d = Different of value in each group, ${\bar{\text{x}}_{\text{d}}}$ = Different of mean in each group, σ^2^_d_ = Different of variance in each group, Z_α/2_ = Standard normal variate for level of significance, Z_β_ = Standard normal variate for power.

We calculated and found that the sample size for our study was 12.51 samples, confirming that this study has recruited enough samples for the experimentation.

### Blood sample collection

Two ml of EDTA blood was extracted from all patients. All samples were collected in 4 × 6 ml K3 EDTA tubes (VACUETTE, Greiner Bio-One GmbH, Kremsmünster, Austria) and provided by King Chulalongkorn Memorial Hospital. All samples were centrifuged for 15 min at 3000 rpm to separate the WBC layer (buffy coat). Then, 100 μl of the WBC layer was transferred to a 1.5 ml Eppendorf tube. The cells were washed with 1 ml PBS for 15 min at 1700 rpm 16 °C. All subjects participating in blood collection were given a self-administered questionnaire to collect their medical history, which was carefully recorded. All samples were obtained under a research protocol approved by the Ethics Committee, Faculty of Medicine, Chulalongkorn University, Thailand (approval number: IRB 034/59. The collection of blood samples from all participants was performed based on the World Health Organization (WHO) guidelines. This study was conducted in accordance with the Declaration of Helsinki. Signed informed consent was obtained.

### RNA extraction

The WBC pellets were mixed with 500 μl of TRIzol reagent (ThermoFisher Scientific, MA, USA) and incubated at room temperature for 10 min, then 200 μl of chloroform was added and incubated at room temperature for 3 min. The sample was then separated into three phases by centrifugation at 8500 rpm at 4 °C for 15 min. The colorless upper aqueous phase was transferred to a new RNA 1.5 ml Eppendorf tube, supplemented with 4 μl of glycogen (20 mg/mL) and 500 μl of 100% isopropanol, incubated for 10 min at room temperature, then centrifuged at 8500 rpm at 4 °C for 10 min. Supernatants of the centrifuged tubes were discarded. The RNA pellets were washed with 1 ml of 75% ethanol and mixed by vortexing. The samples were then centrifuged at 7000 rpm at 4 °C for 5 min. The supernatants were discarded and the RNA pellets were dried by vacuum for 8 min. The RNA pellets were resuspended with 20 μl of DEPC water. Finally, RNA concentration and integrity were evaluated by Nanodrop and bioanalyzer.

### Complementary DNA (cDNA) synthesis

The cDNA was synthesized using RevertAid First Strand cDNA Synthesis (Thermo Scientific) following the manufacturer’s protocol. Briefly, the amount of RNA templates was adjusted into 0.5–1 μg in each reaction. The samples were added to the 1 μl of oligo dT and incubated at 65 °C for 5 min. The following solutions including primer 1 μl, nuclease-free water up to 12 μl, 5X reaction buffer 4 μl, Ribolock RNAse inhibitor 1 μl, 10 mM dNTP mix 2 μl, RevertAid M-MuLV RT 1 μl were added to the samples. After mixing and brief centrifuging, the samples were incubated for 60 min at 42 °C followed by 5 min at 70 °C. The product of the first strand cDNA synthesis can be used directly in PCR or quantitative real-time PCR.

### Primer design and preparation

Primers were designed using reference gene sequences from NCBI database including CR542168.1 for *FKBP1A*, NM_001130081.3 for *PLD1* and NM_001330675.2 for *PSMA4*. The primers were synthesized by U2Bio. The details of the primer sequence, melting temperature, and product length are described in Table 2. Prior to quantitative PCR, conventional PCR and electrophoresis for finding optimal temperature for each primer was conducted.

**Table 2:** Details of forward and reverse primer sequences of three candidate genes which used qRT-PCR analysis including *FKBP1A, PLD1,* and *PSMA4*.

Gene	Forward	Reverse	Tm	Product size (bp)
*FKBP1A*	5′-CGTGGTGCACTACACCGGG-3′	5′-ACCCACACTCATCTGGGCAACCC-3′	60.5	100
*PLD1*	5′-CATAAAGGTGATGAGACACCCGG-3′	5′-TCTGTGCTCATTGTCGTCCCACC-3′	59.8	102
*PSMA4*	5′-CATTGGCTGGGATAAGCACTATGGC-3′	5′-CAACATTGACACAGCTGCAGCGC-3′	60.4	70

### Quantitative real-time PCR (qRT-PCR)

The quantitative real-time PCR contained 10 µl SensiFast Lo-ROX reagent (Bioline), 0.8 µl of forward and reverse mixture primers, 1 µl of cDNA sample, 0.1 µl of Taq polymerase, and 8.1 µl distilled water in a total volume of 20 µl. The reactions were operated by QuantStudio 5 (Thermo Fisher Scientific) following with the manufacturer’s protocol. The amplification conditions were as follows: denaturation at 95 °C for 2 min with 40 cycles, annealing at 60 °C, respectively for 30 s. The positive signals from the amplified product were detected at the end of the annealing step. Duplications were in all samples. In this study, glyceraldehyde 3-phosphate dehydrogenase (GAPDH) was selected as the housekeeping gene or the reference gene. GAPDH gene was analyzed alongside all candidate genes.

The amplification results were calculated with the following formula:

$$\Delta \Delta {\text{Ct}} = \Delta {\text{Ct}}\;\left( {\text{a target sample}} \right) - \Delta {\text{Ct}}\;\left( {\text{a reference sample}} \right)$$

The results were then represented in the folds of change (the equation is in the form of 2^−ΔΔCt^) of the candidate gene expression in the sample against the reference sample^30^.

### ROC analysis

The Receiver Operating Characteristic (ROC) curves were calculated with MedCalc program version 22.009 (Belgium). Ct values of each candidate gene were entered into the dataset. The sensitivity, specificity, and area under curve (AUC) were calculated for all the datasets.

### Statistical analysis

The box plot graph and summary of the dataset (including t-test results of Ct mean of candidate gene) were analyzed with GraphPad Prism version 8.4.3. (MA, USA). The *p-*value < 0.05 was the cut-off for each analysis calculated by unpaired t-tests.

### Ethics approval

All samples were obtained under a protocol approved by the Ethics Committee, Faculty of Medicine, Chulalongkorn University, Thailand (approval number: IRB 034/59). The blood sample collection from all participants was performed in accordance with the WHO guidelines. This study was conducted in accordance with the Declaration of Helsinki. The participants provided their written informed consent to participate in this study.

### Supplementary Information

Supplementary Information.

## Data Availability

Data analyzed and calculated in this study are available from the corresponding author upon reasonable request.

## Abbreviations

cDNA: Complementary DNA
qRT-PCR: Quantitative Real-time PCR
PBS: Phosphate buffer saline
ROC: Receiver operating characteristic
WBC: White blood cell
EDTA: Ethylene diamine tetraacetic acid
MRI: Magnetic resonance imaging
CT: Computed tomography
WHO: World Health Organization
GEO: Gene Expression Omnibus
DEPC: Diethyl pyrocarbonate
CD: Cluster of differentiation

## References

[1] Rahib L, Wehner MR, Matrisian LM, Nead KT (2021). Estimated projection of US cancer incidence and death to 2040. JAMA Netw. Open.

[2] Sung H, Ferlay J, Siegel RL (2021). Global cancer statistics 2020: GLOBOCAN estimates of incidence and mortality worldwide for 36 cancers in 185 countries. CA Cancer J. Clin..

[3] Siegel RL, Miller KD, Jemal A (2018). Cancer statistics, 2018. CA Cancer J. Clin..

[4] Kleeff J, Korc M, Apte M (2016). Pancreatic cancer. Nat. Rev. Dis. Primers.

[5] Mizrahi JD, Surana R, Valle JW, Shroff RT (2020). Pancreatic cancer. The Lancet.

[6] Conroy T, Bachet J-B, Ayav A (2016). Current standards and new innovative approaches for treatment of pancreatic cancer. Eur. J. Cancer.

[7] Gudjonsson B (2002). Survival statistics gone awry: Pancreatic cancer, a case in point. J. Clin. Gastroenterol..

[8] Matsubayashi H, Ishiwatari H, Sasaki K, Uesaka K, Ono H (2020). Detecting early pancreatic cancer: Current problems and future prospects. Gut Liver.

[9] Ilic M, Ilic I (2016). Epidemiology of pancreatic cancer. World J. Gastroenterol..

[10] Latenstein AE, Van Roessel S, Van Der Geest LG (2020). Conditional survival after resection for pancreatic cancer: A population-based study and prediction model. Ann. Surg. Oncol..

[11] Yang J, Xu R, Wang C, Qiu J, Ren B, You L (2021). Early screening and diagnosis strategies of pancreatic cancer: A comprehensive review. Cancer Commun..

[12] Kato S, Honda K (2020). Use of biomarkers and imaging for early detection of pancreatic cancer. Cancers.

[13] Elbanna KY, Jang H-J, Kim TK (2020). Imaging diagnosis and staging of pancreatic ductal adenocarcinoma: A comprehensive review. Insights Imaging.

[14] Wu H, Ou S, Zhang H (2022). Advances in biomarkers and techniques for pancreatic cancer diagnosis. Cancer Cell Int..

[15] Ballehaninna UK, Chamberlain RS (2012). The clinical utility of serum CA 19–9 in the diagnosis, prognosis and management of pancreatic adenocarcinoma: An evidence based appraisal. J. Gastrointest. Oncol..

[16] Tuncer İ, Dülger H, Uygan İ, Öztürk M, Kotan Ç, Sekeroglu R (2004). Comparison of serum cytokeratin-18, CEA and CA 19–9 levels in esophageal and gastric cancers. Eastern J. Med..

[17] Vukobrat-Bijedic Z, Husic-Selimovic A, Sofic A (2013). Cancer antigens (CEA and CA 19–9) as markers of advanced stage of colorectal carcinoma. Med. Arch..

[18] Chen, Y.-L., Chen, C.-H., Hu, R.-H., Ho, M.-C. & Jeng, Y.-M. Elevated preoperative serum CA19-9 levels in patients with hepatocellular carcinoma is associated with poor prognosis after resection. *Sci. World J.* **2013**, 1–6 (2013). 10.1155/2013/380797 PMC3694498 23843733

[19] Coelho R, Silva M, Rodrigues-Pinto E (2017). CA 19-9 as a marker of survival and a predictor of metastization in cholangiocarcinoma. GE-Port. J. Gastroenterol..

[20] Lertkhachonsuk AA, Buranawongtrakoon S, Lekskul N, Rermluk N, Wee-Stekly WW, Charakorn C (2020). Serum CA19-9, CA-125 and CEA as tumor markers for mucinous ovarian tumors. J. Obstet. Gynaecol. Res..

[21] Kim B, Lee K, Moon T (2009). How do we interpret an elevated carbohydrate antigen 19-9 level in asymptomatic subjects?. Digest. Liver Dis..

[22] Kim JE, Lee KT, Lee JK, Paik SW, Rhee JC, Choi KW (2004). Clinical usefulness of carbohydrate antigen 19-9 as a screening test for pancreatic cancer in an asymptomatic population. J. Gastroenterol. Hepatol..

[23] Mosallaei M, Ehtesham N, Rahimirad S, Saghi M, Vatandoost N, Khosravi S (2022). PBMCs: A new source of diagnostic and prognostic biomarkers. Arch. Physiol. Biochem..

[24] Hamm A, Prenen H, Van Delm W (2016). Tumour-educated circulating monocytes are powerful candidate biomarkers for diagnosis and disease follow-up of colorectal cancer. Gut.

[25] Kaur S, Baine MJ, Jain M, Sasson AR, Batra SK (2012). Early diagnosis of pancreatic cancer: Challenges and new developments. Biomark. Med..

[26] Puttipanyalears C, Denariyakoon S, Angsuwatcharakon P (2021). Quantitative STAU2 measurement in lymphocytes for breast cancer risk assessment. Sci. Rep..

[27] Patarat R, Riku S, Kunadirek P (2021). The expression of FLNA and CLU in PBMCs as a novel screening marker for hepatocellular carcinoma. Sci. Rep..

[28] Arayataweegool A, Srisuttee R, Mahattanasakul P (2019). Head and neck squamous cell carcinoma drives long interspersed element-1 hypomethylation in the peripheral blood mononuclear cells. Oral Dis..

[29] Boonsongserm P, Angsuwatcharakon P, Puttipanyalears C (2019). Tumor-induced DNA methylation in the white blood cells of patients with colorectal cancer. Oncol. Lett..

[30] Rao X, Huang X, Zhou Z, Lin X (2013). An improvement of the 2ˆ (–delta delta CT) method for quantitative real-time polymerase chain reaction data analysis. Biostat. Bioinform. Biomath..

[31] Čelešnik H, Potočnik U (2022). Peripheral blood transcriptome in breast cancer patients as a source of less invasive immune biomarkers for personalized medicine, and implications for triple negative breast cancer. Cancers.

[32] Bin-Alee F, Arayataweegool A, Buranapraditkun S (2021). Transcriptomic analysis of peripheral blood mononuclear cells in head and neck squamous cell carcinoma patients. Oral Dis..

[33] Hosseini A, Mirzaei A, Salimi V (2020). The local and circulating SOX9 as a potential biomarker for the diagnosis of primary bone cancer. J. Bone Oncol..

[34] Denariyakoon S, Puttipanyalears C, Chatamra K, Mutirangura A (2022). Breast cancer sera changes in alu element methylation predict metastatic disease progression. Cancer Diagn. Progn..

[35] Pakvisal N, Kongkavitoon P, Sathitruangsak C (2022). Differential expression of immune-regulatory proteins C5AR1, CLEC4A and NLRP3 on peripheral blood mononuclear cells in early-stage non-small cell lung cancer patients. Sci. Rep..

[36] Wedemeyer WJ, Welker E, Scheraga HA (2002). Proline cis−trans isomerization and protein folding. Biochemistry.

[37] Annett S, Moore G, Robson T (2020). FK506 binding proteins and inflammation related signalling pathways; basic biology, current status and future prospects for pharmacological intervention. Pharmacol. Therap..

[38] Wang T, Li B-Y, Danielson PD (1996). The immunophilin FKBP12 functions as a common inhibitor of the TGFβ family type I receptors. Cell.

[39] Patel D, Dabhi AM, Dmello C (2022). FKBP1A upregulation correlates with poor prognosis and increased metastatic potential of HNSCC. Cell Biol. Int..

[40] Xing M, Wang J, Yang Q (2019). FKBP12 is a predictive biomarker for efficacy of anthracycline-based chemotherapy in breast cancer. Cancer Chemother. Pharmacol..

[41] Li Z, Cui Y, Duan Q (2022). The prognostic significance of FKBP1A and its related immune infiltration in liver hepatocellular carcinoma. Int. J. Mol. Sci..

[42] Cai S, Chen Z, Tang H, Meng S, Tao L, Wang Q (2022). Upregulated FKBP1A suppresses glioblastoma cell growth via apoptosis pathway. Int. J. Mol. Sci..

