# Supplementary material for: The high FKBP1A expression in WBCs as a potential screening biomarker for pancreatic cancer
Source: Sci Rep. 2024 Apr 3;14:7888. doi: 10.1038/s41598-024-58324-z (PMC10991374; doi:10.1038/s41598-024-58324-z)
Supplement: Supplementary file 1 — Supplementary Information. [file 41598_2024_58324_MOESM1_ESM.pdf]

Supplement information 1

The details of the biological function of the candidate genes.

Gene symbol	Gene name	Molecular function	Pathologic function	References	Comment	p-value
FKBP1A	FKBP Prolyl Isomerase 1A	The protein encoded by this gene is a member of the immunophilin protein family, which play a role in immunoregulation and basic cellular processes involving protein folding and trafficking. It interacts with several intracellular signal transduction proteins including type I TGF-beta receptor.	An inactive conformation TGFBR1, the TGF-beta type I serine/threonine kinase receptor, preventing TGF-beta receptor activation in absence of ligand. Recruits SMAD7 to ACVR1B which prevents the association of SMAD2 and SMAD3 with the activin receptor complex,	FKBP12 functions as an adaptor of the Smad7-Smurf1 complex on activin type I receptor. (PMID: 16720724) Yamaguchi T ... Sugino H Journal of molecular endocrinology 2006	Immunological process	2.88E-06
PLD1	Phospholipase D1	This gene encodes a phosphatidylcholine-specific phospholipase which catalyzes the hydrolysis of phosphatidylcholine in order to yield phosphatidic acid and choline. The enzyme may play a role in signal transduction and subcellular trafficking.	Implicated as a critical step in numerous cellular pathways, including signal transduction, membrane trafficking, and the regulation of mitosis.	Congenital valvular defects associated with deleterious mutations in the PLD1 gene. (PMID: 27799408) Ta-Shma A ... Elpeleg O Journal of medical genetics 2017	Cellular signaling	2.82E-05
ZC3HAV1L	Zinc Finger CCCH-Type Containing, Antiviral 1 Like	This antiviral protein inhibits viral replication by recruiting cellular RNA degradation machineries to degrade viral mRNAs. The encoded protein plays an important role in the innate immune response against multiple DNA and RNA viruses.	Antiviral protein which inhibits the replication of viruses by recruiting the cellular RNA degradation machineries to degrade the viral mRNAs.	Structural basis for lack of ADP-ribosyltransferase activity in poly(ADP-ribose) polymerase-13/zinc finger antiviral protein. (PMID: 25635049) Karlberg T ... Schüler H The Journal of biological chemistry 2015	Immunological process	1.16E-04
PHF20L1	PHD Finger Protein 20 Like 1	A component of the MOF histone acetyltransferase protein complex	-	Methyllysine reader plant homeodomain (PHD) finger protein 20-like 1 (PHF20L1) antagonizes DNA (cytosine-5) methyltransferase 1 (DNMT1) proteasomal degradation. (PMID: 24492612) Estève PO ... Pradhan S The Journal of biological chemistry 2014	Structural protein	2.39E-04
PSMA4	Proteasome 20S Subunit Alpha 4	This gene encodes a core alpha subunit of the 20S proteosome, which is a highly ordered ring-shaped structure composed of four rings of 28 non-identical subunits. Proteasomes cleave peptides in an ATP- and ubiquitin-dependent manner.	Component of the 20S core proteasome complex involved in the proteolytic degradation of most intracellular proteins. This complex plays numerous essential roles within the cell by associating with different regulatory particles.	Molecular cloning and sequence analysis of cDNAs for five major subunits of human proteasomes (multi-catalytic proteinase complexes). (PMID: 2025653) Tamura T ... Ichihara A Biochimica et biophysica acta 1991	Protein degradation process	4.50E-04

Supplement information 2

The detail of validation set.

		**Normal**	**Pancreatic cancer**
FKBP1A	Positive	2	7
	Negative	9	0
	Total	11	7

% Accuracy = 88.88889
